# Reply to Volkman et al. Comment on “Fust et al. The Potential Economic Impact of the Updated COVID-19 mRNA Fall 2023 Vaccines in Japan. *Vaccines* 2024, *12*, 434”

**DOI:** 10.3390/vaccines12101175

**Published:** 2024-10-17

**Authors:** Michele Kohli, Keya Joshi, Ekkehard Beck, Yuriko Hagiwara, Nicolas Van de Velde, Ataru Igarashi

**Affiliations:** 1 Quadrant Health Economics Inc., 92 Cottonwood Crescent, Cambridge, ON N1T 2J1, Canada; 2 Moderna, Inc., 325 Binney Street, Cambridge, MA 02142, USA; keya.joshi@modernatx.com (K.J.); ekkehard.beck@modernatx.com (E.B.); nicolas.vandevelde@modernatx.com (N.V.d.V.); 3 Moderna, Inc., 4-1-1 Toranomon, Kamiyacho, Minato Ward, Tokyo 105-6923, Japan; yuriko.hagiwara@modernatx.com; 4 Graduate School of Pharmaceutical Sciences, The University of Tokyo, 7-3-1 Hongo, Bunkyo-ku, Tokyo 113-0033, Japan; atarui1@mac.com

**Keywords:** coronavirus, SARS-CoV-2, Japan, COVID-19, vaccination, cost-effectiveness

In their comment [1], Volkman et al. raise a number of questions about our recent modelling of COVID-19 in Japan and our analysis of the cost-effectiveness of vaccination. We welcome the opportunity to address their comment.

Their first cited concern about our article is that there are overlapping confidence intervals (CIs) for the vaccine effectiveness (VE) of the two mRNA COVID-19 vaccines. The statement is incorrect as they have misunderstood how the data were used in our analysis. The VE estimates for mRNA-1273 were based on data for the Omicron-containing bivalent vaccines from the VERSUS study [2]. They correctly cite that we assumed the VE against hospitalization for the mRNA-1273 vaccine would be 84.9% (95% confidence interval [CI]: 65.7 to 93.3%). For our comparison of the two mRNA vaccines, we used data from a real world study by Kopel et al. powered to differentiate the bivalent versions of mRNA-1273 and BNT162b2 [3]. This study used data from a nation-wide dataset from the United States that integrated electronic health records and pharmacy and medical claims data. Kopel et al. concluded that mRNA-1273 has a statistically significantly higher VE than BNT162b2. They estimated the relative VE (rVE) of mRNA-1273 compared to BNT162b2, defined as 100 × (1 − adjusted hazard ratio (HR) from the analysis), to be 5.1% (95% confidence interval: 3.2–6.9%) for outpatient encounters and 9.8% (95% confidence interval 2.6–16.4%) for hospitalizations. For all of our comparative analyses, we used 84.9% as the VE against hospitalization for mRNA-1273 and used the rVE estimate and the 95% CI to calculate the VE of BNT162b2. Therefore, the initial VE of BNT162b2 was 83.3% for the primary comparative scenario, ranging from 81.9% to 84.5% in alternative scenarios. In all analyses in the manuscript, the VE of BNT162b2 was lower than the 84.9% of mRNA-1273 to be consistent with the statistically significant results of the comparative effectiveness study.

The Kopel et al. study we used for our modelling exercise is a well-designed real-world study comparing bivalent vaccines but, as Volkman et al. note, it is only one study. They cite four additional studies of the VE of the COVID-19 vaccine during the Omicron era [4,5,6,7]. However, it is unclear why these studies were specifically referenced as there are other studies that report comparative VEs for mRNA-1273 and BNT16b2. In the Japanese setting, Ono et al. [8] reported a statistically significantly higher VE for the outcome of COVID-19 for the mRNA-1273 monovalent booster compared to BNT162b2. The comparative risk for the age groups targeted by the National Immunization Program (NIP) were a HR of 0.62 (95% CI: 0.41–0.82) for ages 65–84 years and an HR of 0.64 (95% CI: 0.35–0.93) those ≥85 years.

With regard to the three studies cited by Volkman et al., only one of these studies was designed to compare the different mRNA vaccines [4]. The other two studies were designed to investigate mRNA COVID-19 boosters and only included specific vaccine types as part of their regression analyses or sensitivity analyses [5,6]. It is true that none of these cited studies report statistically significant differences between the mRNA vaccines, however, they may not have been powered to detect the differences. For this reason, it is important to consider meta-analyses that combine the results of studies identified through systematic reviews of the literature across different versions of the vaccine, the number of vaccinations received, the SARS-CoV-2 virus variants, and the settings of the pandemic. A recent meta-analysis combined the results of 24 non-randomized real-world studies comparing ≥2 doses of mRNA-1273 and BNT162b2 in immunocompetent adults ≥50 years of age [9]. Overall, mRNA-1273 was associated with a significantly lower risk of SARS-CoV-2 infection and hospitalization compared to BNT162b2, with relative risks (RRs) of 0.72 (95% CI: 0.64–0.80) and 0.65 (95% CI: 0.53–0.79), respectively. Another meta-analysis combined the results of 17 studies comparing mRNA-1273 with BNT162b2 in immunocompromised individuals ages ≥18 years [10]. mRNA-1273 was associated with a statistically significant reduction in both SARS-CoV-2 infection and hospitalization, with RRs of 0.85 (95% CI: 0.75–0.97) and 0.88 (85% CI: 0.79–0.97), respectively. Volkman et al. cite a fourth study posted on a pre-print server [7], that has now been accepted for publication in *Vaccine* [11]. This study, which compares the VE of a third dose of mRNA-1273 to BNT162b2 in preventing hospitalizations among adults ages ≥65 years in the US, concluded that mRNA-1273 was statistically significantly more effective (hazard ratio of 0.82; 95% CI: 0.69–0.98).

In the manuscript, we present the projected differences in clinical impact between the mRNA-1273 and BNT162b2 given the rVEs of 5.1% for infection and 9.8% for hospitalization in Table 6. These projections are conducted using a dynamic transmission model of COVID-19 in Japan that had been described in detail in a previously published manuscript [12]. This type of model is recommended for infectious diseases by the ISPOR-SMDM Good Research Practices in Modeling Task Force because they account for the non-linear impact of transmission [13]. In other words, these models account for the direct benefit of vaccination to those who receive a vaccine, as well as the indirect benefit of blocking transmission by reducing the number of susceptible individuals in a population. Volkman et al. note that “translated into considerably larger differences in terms of the total number of infections (4%) and hospitalizations (6%) averted”. This is indeed to be expected with this type of modelling because of the indirect benefit of vaccination.

Cost-effectiveness analyses play an important part of policy discussions in many countries, such as Japan, which aim to provide good value for health care money spent on their citizens [14,15,16,17]. The primary comparison in our manuscript was vaccinating with mRNA-1273 versus not vaccinating in Fall 2023 in Japan. This comparison was meant to inform policy discussions under the Japanese NIP about COVID-19 vaccination policies in late 2023. At this time, the unit cost of the vaccines had not been established. The NIP decision makers were interested in assessing the cost-effectiveness of routine vaccination (either once per year or twice per year) against no vaccination, under multiple vaccination price assumptions. Therefore, we conducted an analysis of the economically justifiable prices of COVID-19 vaccines under different assumptions and acceptable cost-effectiveness thresholds. The same process of conducting analyses to understand the range of economically justifiable prices was also carried out for the NIP for past vaccines such as the human papillomavirus vaccines [18]. The majority of the analyses in the manuscript therefore focused on this primary comparison. The secondary comparison of mRNA-1273 versus BNT162b2 is of primary interest to vaccine procurement choice processes. We report the results of all scenario analyses with the range of rVEs comparing mRNA-1273 and BNT162b2 for this primary outcome located in Table 5 in the Appendix. Our analysis demonstrates that the observed differences in VE may have implications for potential price differences between the vaccines.

In our analysis, we assumed that both vaccines have the same adverse event profile based on data available from studies conducted with collaboration with the Centers for Disease Control in the United States. Anaphylaxis rates were assumed to be the same because Klein et al. reported an event rate of 5.0 (95% CI: 3.5–6.9) per million doses with mRNA-1273 and 4.9 (95% CI: 3.2–7.2) per million doses with BNT162b2 [19]. For myocarditis and pericarditis, Shimabukuro et al. reported an event rate of 30.9 (95% CI: 18.3–48.9) per million doses for mRNA-1273 and 31.3 (95% CI: 15.0–57.6) per million doses for BNT162b2 [20]. The CIs for these event rates were truly overlapping, which is why we used the same rates for both vaccines in the comparative analysis.

We agree with Volkman et al. that one of the major public health challenges is public fatigue with COVID-19 and decreasing vaccine uptake rates. A systematic review of discrete choice experiments determined that VE is considered to be the most important vaccine attribute regardless of the population included in the study [21,22]. Given vaccine hesitancy, it continues to be important to quantify both the clinical and economic impact of COVID-19 vaccines compared to no vaccines. To encourage vaccine uptake, it is crucial to emphasize the sustained vaccine effectiveness (VE) and the real benefits of ongoing COVID-19 vaccination. The comparison to no vaccine therefore accounts for the majority of the analyses in our manuscript. While not the primary concern, data on the comparative effectiveness of vaccines and the associated cost-effectiveness is important evidence for those decision makers aiming to make efficient use of limited healthcare budgets.

In conclusion, we disagree with the concerns raised by Volkman et al. about our analysis. The estimate of the difference in VE between the vaccines is conservative relative to robust meta-analyses that have been conducted based on systematic literature reviews. We believe that our article has important messages for public health officials and health care decision makers.
